# Rab35 and its effectors promote formation of tunneling nanotubes in neuronal cells

**DOI:** 10.1038/s41598-020-74013-z

**Published:** 2020-10-08

**Authors:** Shaarvari Bhat, Nina Ljubojevic, Seng Zhu, Mitsunori Fukuda, Arnaud Echard, Chiara Zurzolo

**Affiliations:** 1 Unit of Membrane Traffic and Pathogenesis, UMR3691 CNRS, Institut Pasteur, 28 rue du Dr Roux, 75015 Paris, France; 2 Université Paris-Sud, Université Paris-Saclay, 91405 Orsay, France; 3 Sorbonne Université, ED394-Physiologie, Physiopathologie et Thérapeutique, 75005 Paris, France; 4 Department of Integrative Life Sciences, Graduate School of Life Sciences, Tohoku University, Aobayama, Aoba-ku, Sendai, Miyagi 980-8578 Japan; 5 Membrane Traffic and Cell Division Lab, UMR3691 CNRS, Institut Pasteur, 75015 Paris, France

**Keywords:** Endosomes, Small GTPases, Cell biology, Membrane trafficking

## Abstract

Tunneling nanotubes (TNTs) are F-actin rich structures that connect distant cells, allowing the transport of many cellular components, including vesicles, organelles and molecules. Rab GTPases are the major regulators of vesicle trafficking and also participate in actin cytoskeleton remodelling, therefore, we examined their role in TNTs. Rab35 functions with several proteins that are involved in vesicle trafficking such as ACAP2, MICAL-L1, ARF6 and EHD1, which are known to be involved in neurite outgrowth. Here we show that Rab35 promotes TNT formation and TNT-mediated vesicle transfer in a neuronal cell line. Furthermore, our data indicates that Rab35-GTP, ACAP2, ARF6-GDP and EHD1 act in a cascade mechanism to promote TNT formation. Interestingly, MICAL-L1 overexpression, shown to be necessary for the action of Rab35 on neurite outgrowth, showed no effect on TNTs, indicating that TNT formation and neurite outgrowth may be processed through similar but not identical pathways, further supporting the unique identity of these cellular protrusions.

## Introduction

Tunneling nanotubes (TNTs) are a type of intercellular structures discovered more than a decade ago by Rustom et al. and classified as membrane protrusions that hover above the substrate while connecting two distant cells^1^. Since then, many characteristics have been determined for TNTs that substantially differentiate them from similar structures, e.g. filopodia and cytonemes^2,3^. Their diameter ranges from 50 to 800 nm and they can extend up to a hundred micrometres in length^1,4^. Recently, structural composition of TNTs was characterized as containing mainly straight actin filaments^4^, and in some cases microtubules as well^5^. One of the main features assigned to TNTs which makes them distinguished is that they are open-ended, allowing for various cargoes to transfer between cells. These cargoes include organelles, such as lysosomes^1,6^ and mitochondria^7^, but they have additionally been identified as conduits for micro-RNA^8^, calcium^9^, proteins such as class I MHC receptor^10^, amyloids^6,11^ and many others^12^.

Apart from playing a role in development, as discovered for TNT-like structures present in chick and sea urchin embryos^3,13,14^, their implication in various diseases was tackled in many studies. They were shown to be hijacked and utilized as means of transport by different pathogens, such as viruses like HIV^15,16^, and bacteria^17^. Interestingly, TNTs have been shown to be involved in spreading of amyloid proteins such as prions^18^, alpha synuclein^19,20^, huntingtin^21^ etc. from diseased to healthy neighbouring cells, and therefore suggested to have a role in the progression of different neurodegenerative diseases^22^. Additionally, TNTs have been found in several types of cancer cells where they have been correlated with cancer progression^23–25^.

Two major mechanisms for TNT formation have been hypothesized: (1) subsequent to the dislodgement of two cells in close proximity, a thin thread of membrane remains that forms a TNT or (2) actin-driven protrusion formation, where an initial filopodia-like protrusion reaches the other cell and fuses with it, giving rise to a functional TNT^26–29^.

In neuronal CAD cells, the most prevalent mechanism is the actin-driven one^26^. Interestingly, in these cells an actin bundling and capping protein Eps8 positively regulated TNTs via its actin bundling activity^30^. Recently it was also reported that in these cells upon Arp2/3 inhibition there is an increase in TNTs and the vesicle transfer they conduct^4^.

Several studies explored diverse molecules and their respective roles in TNT formation in different cell models^2,31^. In addition to actin polymerization, membrane recycling also plays a role in the protrusion formation^32,33^. Vesicle trafficking is a process that regulates membrane compartments and it includes exocytosis and endocytosis^34^. Upon endocytosis certain proteins are directed towards the endosomal recycling pathway that involves the return of the vesicles and receptors back to the plasma membrane^35^. Thus, membrane recycling process is regulating plasma membrane composition by maintaining the balance between uptake and recycling. This in turn contributes to several cellular processes such as cytokinesis, cell migration, polarization and signal transduction^34^. Interestingly, recycling of vesicular membranes was found to be important in protrusion formation^32,33,36^.

Rab proteins are small GTPases known to be master regulators of cellular processes which play a major role in vesicle trafficking^37^. Of interest, Rab GTPases have been shown to have a role in cytokinesis^38,39^ and in the formation of different types of protrusions like filopodia, cilia and neurites^33,37,40^. Specifically, Rab8 was shown to promote the formation of macropinosome protrusion by regulating membrane recycling, whose downregulation on the other hand had a negative impact on lamellipodia formation^41^. Both Rab8 and Rab11 participated in the process of ciliogenesis, through the action of Rabin8, possibly also enhancing vesicular recycling^42^. In addition, Rab35, a master regulator of membrane recycling^43,44^, was also shown to be involved in ciliogenesis^40^, as well as in the formation of neurites, the initial cellular protrusions giving rise to axons and dendrites^33,45^.

Considering the significant amount of literature supporting the role of diverse Rab GTPases in membrane protrusion formation^32,33,40–42^, we hypothesized that some of them might be involved in TNT regulation. Therefore, in a screen to assess the role of Rab GTPases in TNTs we revealed that Rab8a, Rab11a and Rab35 had a positive impact on contact-dependent vesicle transfer^32^. Further analysis of the action of Rab11a and Rab8a revealed that they acted in a cascade pathway that activated VAMP3 to positively regulate TNTs^32^, sustaining previous reports^46^. In contrast, how Rab35 might play a role in TNT formation was not explored.

Rab35 is involved in fast endocytic recycling, cytokinesis and cilium formation^38,40,47–49^. Of specific interest, it was shown that it directly interacts with an effector protein called MICAL-L1 (molecules interacting with CasL-like 1) to target it to the recycling endosomes^50,51^ to promote neurite outgrowth^33,52^. Interestingly, MICAL- L1 was shown in the same study to directly recruit EHD1 (EH domain-containing 1) to the same compartment^33^. EHD1 has been known to promote trafficking from recycling endosomes to the plasma membrane by localizing at recycling endosomes^53^. Since endocytic recycling could be crucial to supplying membranes and/or proteins to neurite tips to enable their outward growth^54^, the authors proposed that EHD1 would promote this process by facilitating fission of vesicles targeted to neurite tips from recycling endosomes^33^. Interestingly, the authors also showed that Rab35 directly interacts with ACAP2 (ARF GAP with coiled coil, ANK repeat and PH domain), also called Centaurin β2, to promote the recruitment of EHD1^33^. ACAP2, being a GTPase-activating protein (GAP) of ARF6, inhibits ARF6 which might lead to an increase in the production of phosphatidylinositol-4-phosphate (PI4P)^55,56^. The elevated levels of PI4P will therefore enable more binding of EHD1 to the recycling endosomes^33^. Conversely, ARF6-GTP (the active form) was found to directly interact with EPI64, a GAP of Rab35 and therefore inactivates Rab35^49,57,58^. Interestingly, ARF6-GDP was also demonstrated to participate in the process of neurite outgrowth along Rab35-GTP^33^. Therefore, there seems to be a bistable switch between the active/inactive state of Rab35 and ARF6, where they act oppositely on each other^48^.

Similar to neurite outgrowth, TNT formation in neuronal cells involves both recycling of the membrane^32,59^ and cytoskeletal assembly^30^, thus we postulated that Rab35 may act through a similar pathway as for neurite outgrowth in regulating TNTs. By overexpression/downregulation of Rab35 and different downstream effectors, we found that Rab35 positively regulates TNTs and vesicle transfer between connected cells, via its active GTP-bound form. Importantly, we showed that Rab35 works through ACAP2 and ARF6-GDP, but does not require MICAL-L1. We demonstrated that Rab35, ACAP2 and ARF6-GDP act upstream of EHD1 to promote TNT formation. Over all, this data demonstrates that activation of vesicle recycling through Rab35 is necessary for TNT formation, in a similar but distinct pathway from the one shown to be activated in the case of neurite elongation, where MICAL-L1 is required. This indicates that the mechanisms of formation of TNTs and neurite protrusion are different.

## Results

### Rab35 promotes the formation of functional TNTs

**Figure 1:**
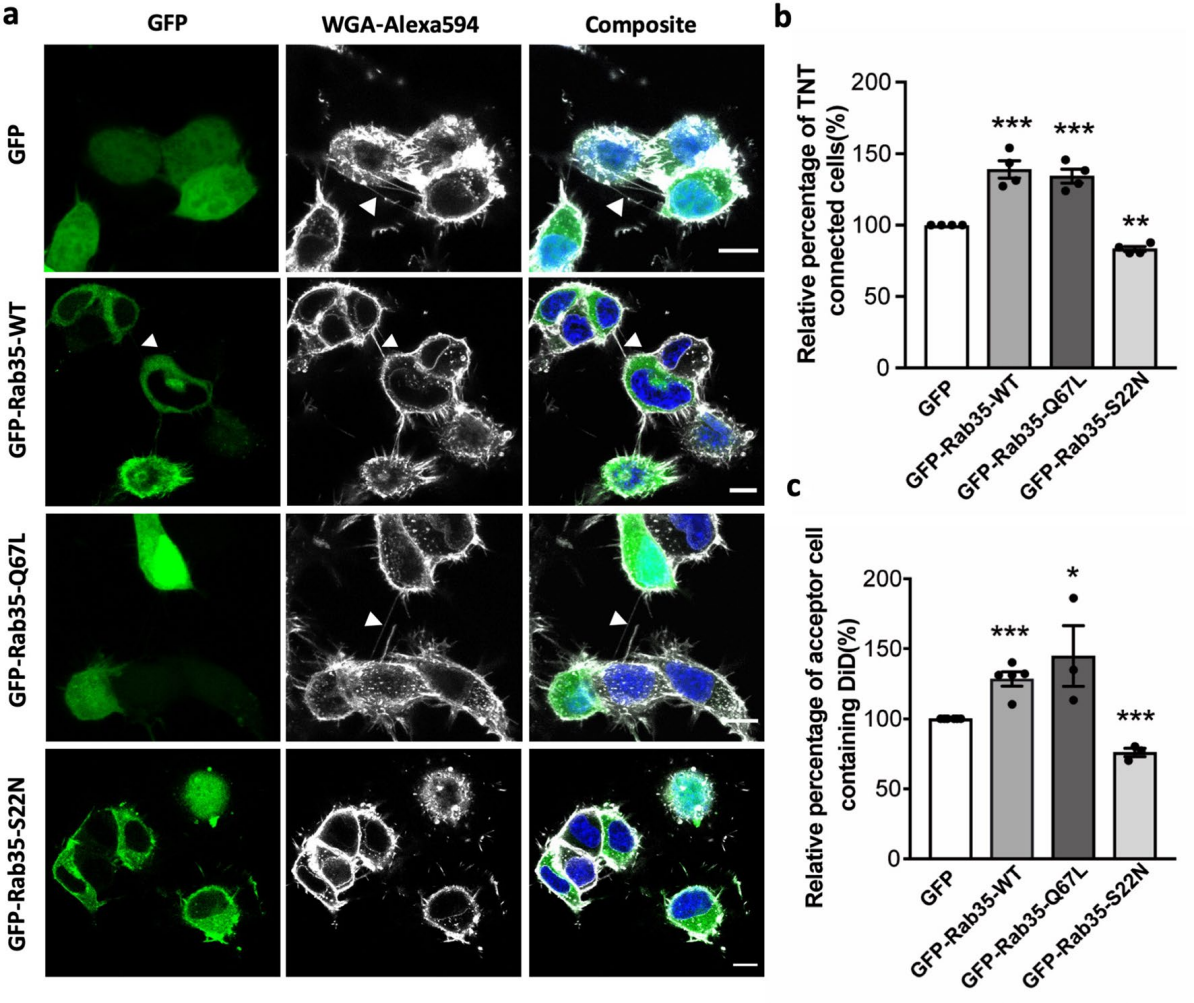
Rab35 promotes the formation of functional TNTs: (**a**) Confocal images of cells transfected with GFP, GFP-Rab35-WT, GFP-Rab35-Q67L, GFP-Rab35-S22N. Arrows indicate TNTs. (**b**) Bar graphs representing the relative percentage of TNT-connected cells described in (**a**) (GFP = 100%, GFP-Rab35-WT = 139 ± 9.9%, GFP-Rab35-Q67L = 134.4 ± 8.7%, GFP-Rab35-S22N = 83.3 ± 4.2%). (**c**) Bar graph showing the percentage of acceptor cells containing DiD-labelled vesicles from the co-cultures, where donor cells were transfected with GFP, GFP-Rab35-WT, GFP-Rab35-Q67L, GFP-Rab35-S22N (GFP = 100%, GFP-Rab35-WT = 128.5 ± 5.0%, GFP-Rab35-Q67L = 144.9 ± 21.6%, GFP-Rab35-S22N = 76.0 ± 3.0%). All graphs from at least three independent experiments and show mean ± SEM. (ns, not significant; *P < 0.05, **P < 0.01, ***P < 0.001; by one-way ANOVA with Tukey’s multiple comparison test). All bar graphs were analysed and represented using Graph Pad Prism version 7. Scale bars 10 µm.

Based on the effect of Rab protein overexpression to affect TNT-mediated vesicle transfer in CAD cells, we previously carried a screen of 41 Rabs and identified Rab35 as a positive regulator of TNT formation^32^. However, whether the activation of Rab35 is important and which Rab35 effectors are involved are unknown. To address these questions, we first transfected either the wild type (WT), active (Q67L) or inactive (S22N) mutant of Rab35 tagged with GFP in neuronal CAD cells. After transfection, cells were cultured for 16 h and then fixed to quantify the number of TNT-connected cells. Because no specific marker of TNTs is currently available, TNTs were identified in culture between cells labelled with WGA (wheat germ agglutinin) and/or phalloidin, as previously shown^60^, according to the current definition of TNTs: membranous connections between cells that do not contact the substrate^1,60^ (see Movie S1 and “Materials and methods” for a more detailed description). Overexpression of GFP-Rab35-WT and GFP-Rab35-Q67L resulted in an increase in number of TNT-connected cells (Fig. 1a,b), while overexpression of the dominant negative mutant of GFP-Rab35-S22N led to a decrease in the number of TNT-connected cells (Fig. 1a,b). TNTs formed between GFP-Rab35-WT cells contained the Rab35-WT protein within the actin-supported TNT, but also DiD-stained vesicles (Fig. S1), suggesting the ability of these TNTs to transfer cargo and the relevance of Rab35-WT in TNT formation. In order to examine and quantitatively analyse whether the intercellular connections induced by Rab35 identified by morphological criteria were functional TNTs, we next measured the transfer of DiD-labelled vesicles between two populations of cells in co-culture as previously described^30,60^. Briefly, donor cells (transfected with either GFP–Rab35-WT, active (Q67L) or inactive (S22N)) were loaded with a fluorescent membrane dye (Vybrant DiD) to label all internal vesicles, mixed at a 1:1 ratio with acceptor cells (transfected with mCherry-H2B, to distinguish them from donors) and co-cultured for 16 h. As controls, donor cells were transfected only with GFP empty vectors (see Fig. S2). The transfer was measured by flow cytometry and the corresponding gating strategy is shown in Fig. S3. Additionally, secretion-based transfer was quantified (Fig. S2, see “Materials and methods” for more details). To visualize the co-culture setup, confocal images were acquired and represented in Fig. S4. Overexpression of GFP–Rab35-WT and Q67L significantly increased the transfer of DiD-labelled vesicles, as measured by flow cytometry and visualized by microscopy (Fig. 1c, Figs. S3a, S4). In contrast, overexpression of the dominant negative mutant of GFP-Rab35-S22N showed a decrease in vesicle transfer between the cells as compared to the control (Fig. 1c, Fig. S3a). Secretion-based transfer was much lower than total transfer (Fig. S3a), suggesting that it had little to no impact on the transfer of the vesicles in our cells. Indeed, increase/decrease in cell–cell contact-based transfer is confirmed by the same trend in the amount of TNT-connected cells. Thus, this data shows that active Rab35 promotes the formation of TNTs and leads to an increase in cargo transfer between cells indicating that these TNTs are functional.

### ACAP2 promotes the formation of functional TNTs

In order to understand how Rab35 regulates TNT formation, we decided to look at several downstream effectors. First, we assessed the role of the Rab35 effector ACAP2/centaurin-β2 which was shown to be recruited by Rab35 and to act downstream of it in promoting neurite outgrowth^33^.

**Figure 2:**
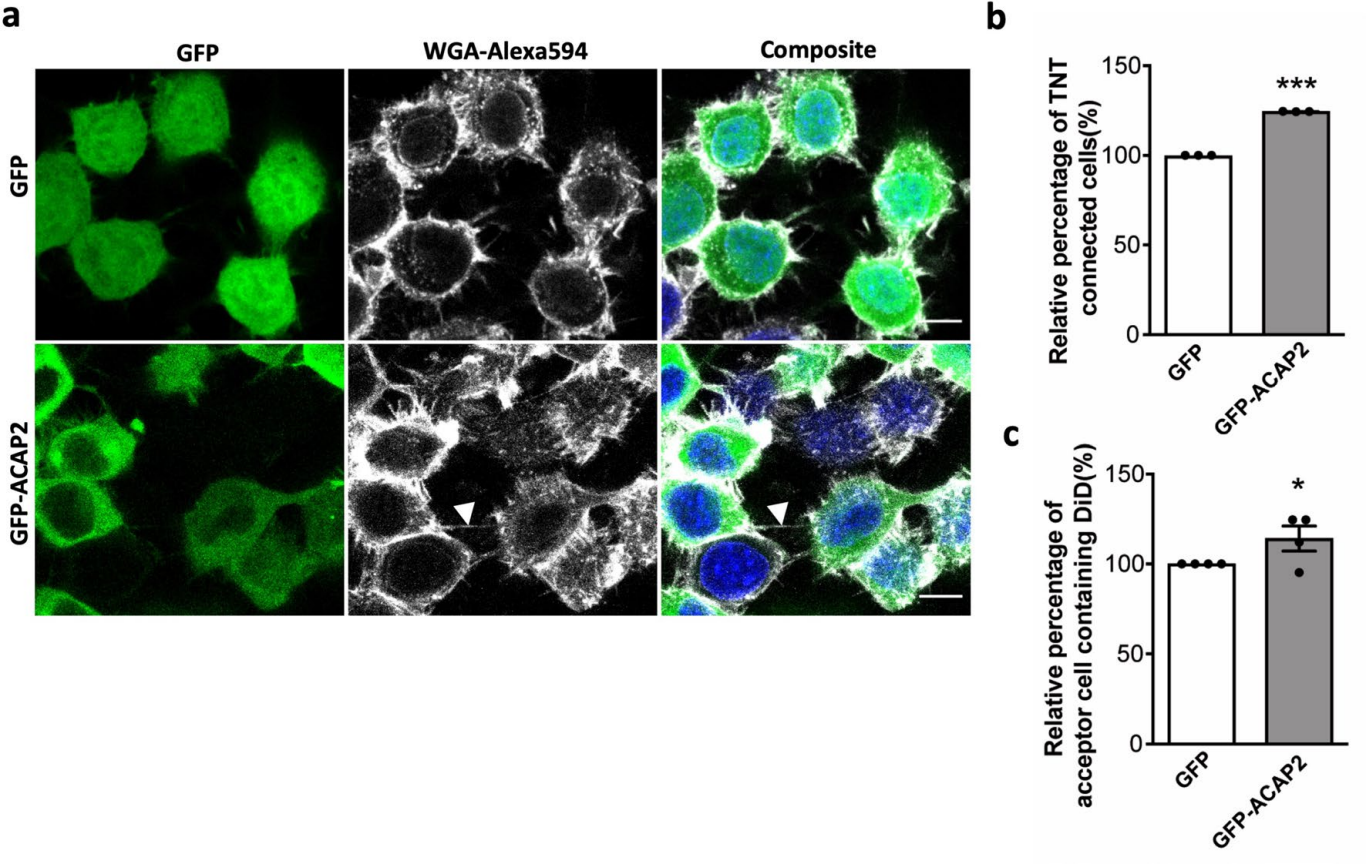
ACAP2 promotes the formation of functional TNTs: (**a**) Confocal images of cells transfected with GFP, GFP-ACAP2-WT. Arrows indicate TNTs. (**b**) Bar graphs representing the relative percentage of TNT-connected cells described in (**a**) (GFP = 100%, GFP-ACAP2 = 124.4 ± 0.1%). (**c**) Bar graph showing the relative percentage of acceptor cells containing DiD-labelled vesicles from the co-cultures, where donor cells were transfected with GFP, GFP-ACAP2 (GFP = 100%, GFP-ACAP2 = 114.1 ± 6.9%). All graphs from at least three independent experiments and show mean ± SEM. (ns, not significant; *P < 0.05, **P < 0.01, ***P < 0.001; by unpaired Student’s t-test). All bar graphs were analysed and represented using Graph Pad Prism version 7. Scale bars 10 µm.

By using the same methods described before, donor CAD cells were transfected with GFP (control) and GFP-ACAP2-WT for 24 h and re-plated for 16 h as single cell populations to analyse the percentage of TNT-connected cells or mixed in co-culture with mCherry-H2B acceptor cells to analyse vesicle transfer by flow cytometry. Overexpression of GFP-ACAP2-WT resulted in an increase in both the number of TNT-connected cells (Fig. 2a,b), where TNTs indeed contained actin and GFP-ACAP2 (Fig. S1), and vesicle transfer between the cells (Fig. 2c, Figs. S3b, S4). This data suggested that Rab35 may regulate TNT formation between the cells through ACAP2.

### Inactivation of ARF6 leads to an increase in TNTs

**Figure 3:**
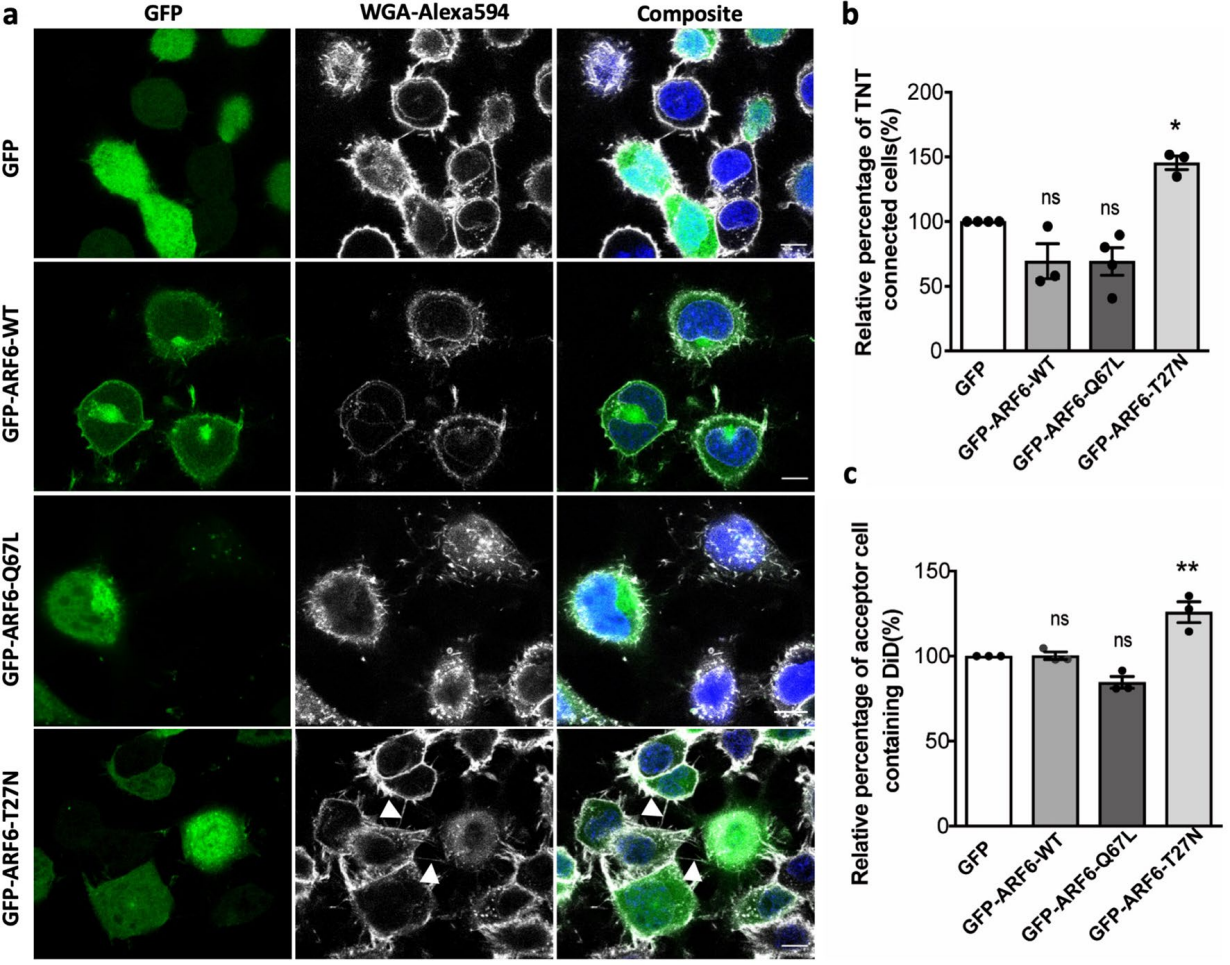
Inactivation of ARF6 leads to an increase in TNTs: (**a**) Confocal images of cells transfected with GFP, GFP-ARF6-WT, GFP-ARF6-Q67L, GFP-ARF6-T27N. Arrows indicate TNTs. (**b**) Bar graphs representing the relative percentage of TNT-connected cells described in (**a**) (GFP = 100%, GFP-ARF6-WT = 69.4 ± 4.6%, GFP-ARF6-Q67L = 69.2 ± 3.7%, GFP-ARF6-T27N = 142.2 ± 4.7%). (**c**) Bar graph showing the relative percentage of acceptor cells containing DiD-labelled vesicles from the co-cultures, where donor cells were transfected with GFP, GFP-ARF6-WT, GFP-ARF6-Q67L, GFP-ARF6-T27N (GFP = 100%, GFP-ARF6-WT = 100.2 ± 2.2%, GFP-ARF6-Q67L = 84.1 ± 3.5%, GFP-ARF6-T27N = 125.8 ± 6.1%). All graphs from at least three independent experiments and show mean ± SEM. (ns, not significant; *P < 0.05, **P < 0.01, ***P < 0.001; by one- way ANOVA with Tukey’s multiple comparison post-test). All bar graphs were analysed and represented using Graph Pad Prism version 7. Scale bars 10 µm.

ACAP2 is a GAP for ARF6, and inactivation of ARF6 by ACAP2 is required for successful neurite outgrowth of PC12 cells^45^. We hypothesized that a similar mechanism could be involved in TNT formation. To test this idea, we first analysed the effect of ARF6 mutants on TNTs. We transfected cells with either GFP-ARF6-WT (wild type), GFP-ARF6-Q67L (active, GTP bound) and GFP-ARF6-T27N (dominant negative, GDP bound) mutants and measured the number of TNTs as described above. Overexpression of GFP-ARF6-WT and GFP-ARF6-Q67L had no impact on the number of TNT-connected cells (Fig. 3a,b), nor on vesicle transfer between the cells (Fig. 3c). Furthermore, the overexpression of inactive GFP-ARF6-T27N in the cells demonstrated an increase in the number of TNT-connected cells (Fig. 3a,b), whose TNTs were supported by actin and even contained DiD-stained vesicles; overexpression also increased vesicle transfer between cells, as quantified by flow cytometry and visualized by confocal microscopy (Fig. 3c, Figs. S3c, S4). Overall, this data showed that the inactivation of ARF6 (by overexpressing its dominant negative mutant) increases the formation of functional TNTs, indicating that ARF6 in its inactive state promotes TNT formation. These results are consistent with the observation that the overexpression of the ARF6 GAP ACAP2 (Fig. 2) increases TNT formation.

### EHD1 is required for TNT formation

**Figure 4:**
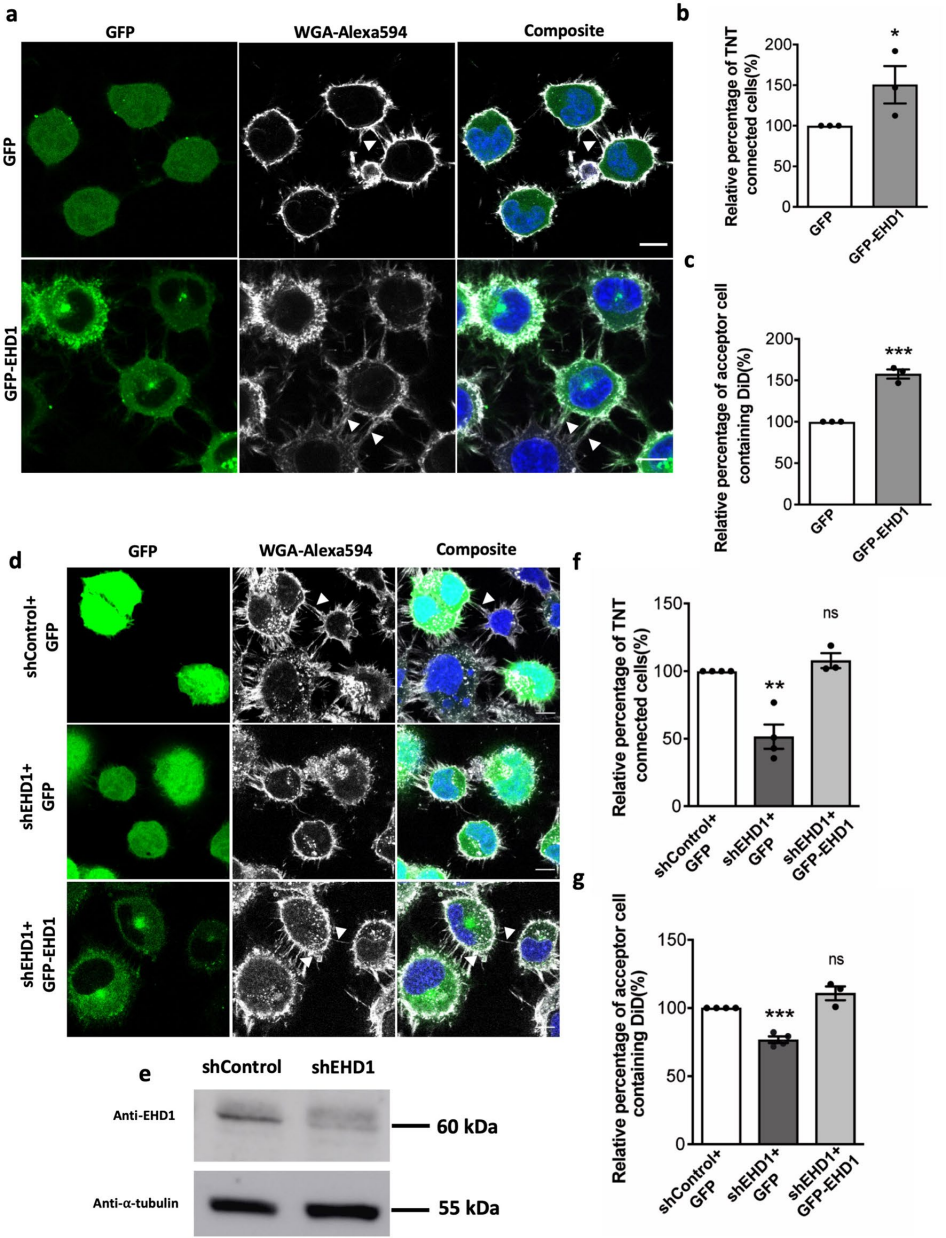
EHD1 is required for TNT formation: (**a**) Confocal images of cells transfected with GFP, GFP-EHD1. Arrows indicate TNTs. (**b**) Bar graphs representing the relative percentage of TNT-connected cells described in (**a**) (GFP = 100%, GFP-EHD1 = 150.5 ± 23%). (**c**) Bar graph showing the relative percentage of acceptor cells containing DiD-labelled vesicles from the co-cultures, where donor cells were transfected with GFP, GFP-EHD1 (GFP = 100%, GFP-EHD1 = 157.6 ± 5.5%). All graphs from three independent experiments and show mean ± SEM. (ns, not significant; *P < 0.05, **P < 0.01, ***P < 0.001; by unpaired Student’s t-test); (**d**) Confocal images of cells transfected with shControl + GFP, shEHD1 + GFP, shEHD1 + GFP-EHD1. Arrows indicate TNTs. (**e**) Western blot of cells transfected with shRNA non-targeting (shCTL) or targeting EHD1 (shEHD1), showing the expression of EHD1 and α-tubulin as loading control. (**f**) Bar graphs representing the relative percentage of TNT-connected cells described in (**a**) (shControl + GFP = 100%, shEHD1 + GFP = 51.7 ± 12.7%, shEHD1 + GFP-EHD1 = 110.5 ± 8.2%). (**g**) Bar graph showing the relative percentage of acceptor cells containing DiD- labelled vesicles from the co-cultures, where donor cells were transfected with shControl + GFP, shEHD1 + GFP, shEHD1 + GFP-EHD1 (shControl + GFP = 100%, shEHD1 + GFP = 51.5 ± 9.0%, shEHD1 + GFP-EHD1 = 107.9 ± 5.6%). All graphs from at least three independent experiments and shows mean ± SEM. (ns, not significant; *P < 0.05, **P < 0.01, ***P < 0.001; by one-way ANOVA with Tukey’s multiple comparison test). All bar graphs were analysed and represented using Graph Pad Prism version 7. Scale bars 10 µm.

Considering that active ARF6 converts PI4P to PI(4,5)P2 by activation of PIP5 kinase^61^, it was speculated that inactivation of ARF6 might be required to maintain PI4P which in turn can recruit EHD1 that has an essential role in promoting neurite outgrowth following Rab35 activation^33^. Following this line of thought, we next tested the role of EHD1 in TNTs. Overexpression of the wild type form of EHD1 showed an increase in the number of TNT-connected cells (Fig. 4a,b); these TNTs contained throughout their length not only actin, but the EHD1 protein, as well as DiD vesicles, suggesting the importance of EHD1 in TNT formation and the ability of TNTs to transfer cargo (Fig. S1). The amount of vesicles transferred between the cells was also increased, as quantified by flow cytometry and represented by confocal microscopy (Fig. 4c, Figs. S3d, S4).

By using shRNA against EHD1 we observed a decrease in the expression of endogenous EHD1 (Fig. 4e, Fig. S5). Of importance, these cells showed a decrease in the number of TNT-connected cells (Fig. 4d,f) and in vesicle transfer between the cells (Fig. 4g, Fig. S3e). In addition, by re-expressing EHD1 in cells depleted of endogenous EHD1, we could rescue the number of TNT-connected cells (Fig. 4d,f) and the transfer rate (measured by flow cytometry as % of acceptor cells containing transferred vesicles) (Fig. 4g, Fig. S3e). Altogether, this data indicates that EHD1 is required for the formation of functional TNTs.

One important control for the specificity of this pathway in TNT formation, was to check whether Rab35 and its effectors were able to impact filopodia formation. Indeed, it has been clearly shown in CAD and other neuronal cells, that TNTs and filopodia are distinct structures^4^ for which the mechanism of formation is different^30^. Interestingly, overexpressing either GFP-ARF6-T27N, GFP-ACAP2, GFP-Rab35 or GFP-EHD1 showed no effect on the number of attached filopodia (Fig. S6a,b), strengthening the specificity of this pathway for TNTs.

### ARF6-GDP, ACAP2 and Rab35 act upstream of EHD1 to regulate TNTs

**Figure 5:**
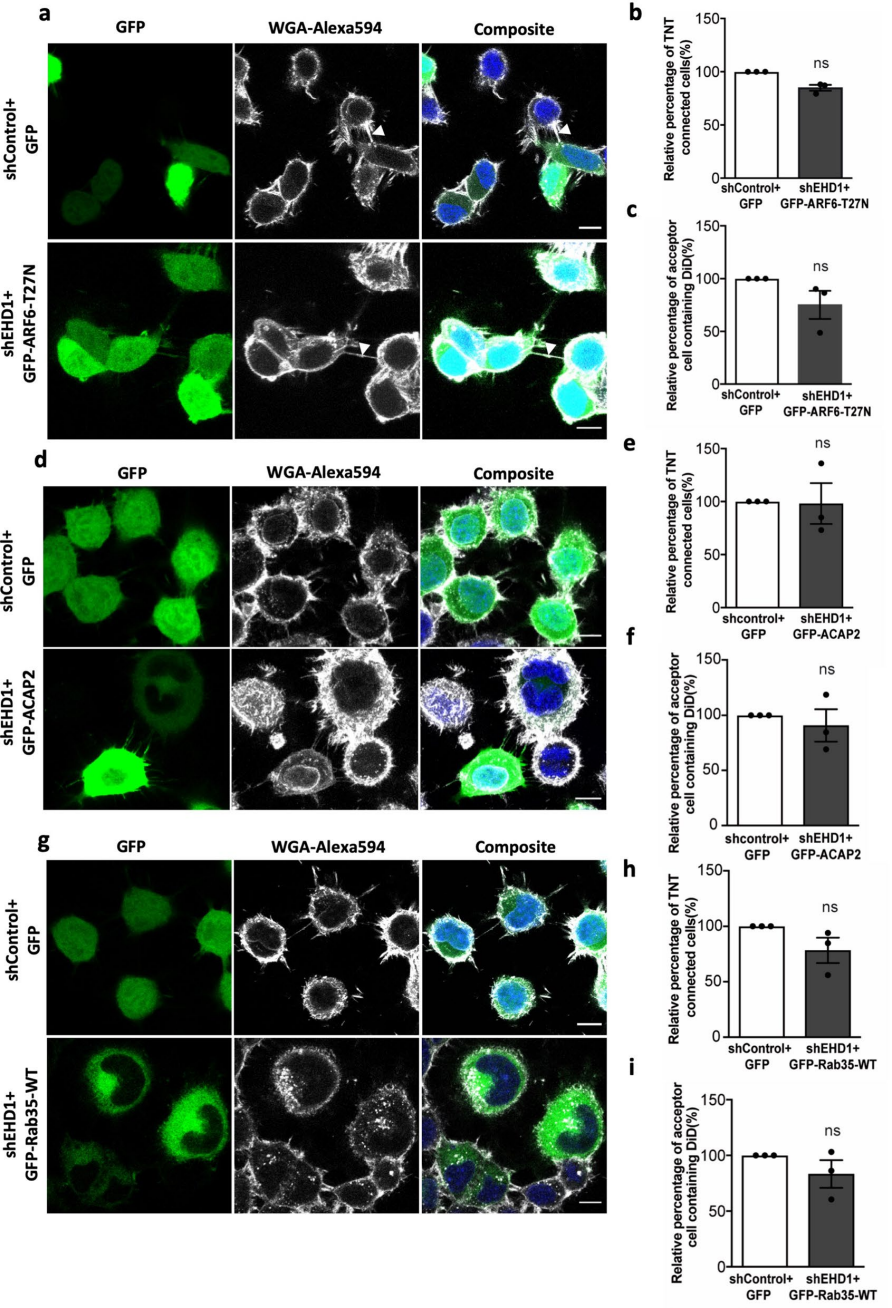
ARF6-GDP, ACAP2 and Rab35 act upstream of EHD1 to regulate TNTs: (**a**) Confocal images of cells transfected with shControl + GFP, shEHD1 + GFP-ARF6-T27N. Arrows indicate TNTs. (**b**) Bar graphs representing the relative percentage of TNT-connected cells described in (**a**) (shControl + GFP = 100%, shEHD1 + GFP-ARF6-T27N = 83.7 ± 4.4%). (**c**) Bar graph showing the relative percentage of acceptor cells containing DiD-labelled vesicles from the co-cultures, where donor cells were transfected with shControl + GFP, shEHD1 + GFP-ARF6-T27N (shControl + GFP = 100%, shEHD1 + GFP-ARF6-T27N = 75.1 ± 13.3%). (**d**) Confocal images of cells transfected with shControl + GFP, shEHD1 + GFP-ACAP2. Arrows indicate TNTs. (**e**) Bar graphs representing the relative percentage of TNT-connected cells described in (**d**) (shControl + GFP = 100%, shEHD1 + GFP-ACAP2 = 98.1 + 19.3%). (**f**) Bar graph showing the relative percentage of acceptor cells containing DiD-labelled vesicles from the co-cultures, where donor cells were transfected with shControl + GFP, shEHD1 + GFP-ACAP2 (shControl + GFP = 100%, shEHD1 + GFP-ACAP2 = 90.8 + 14.6%). (**g**) Confocal images of cells transfected with shControl + GFP, shEHD1 + GFP- Rab35-WT. Arrows indicate TNTs. (**h**) Bar graphs representing the relative percentage of TNT-connected cells described in (**g**) (shControl + GFP = 100%, shEHD1 + GFP-Rab35-WT = 78.3 ± 11.2%). (**i**) Bar graph showing the relative percentage of acceptor cells containing DiD-labelled vesicles from the co-cultures, where donor cells were transfected with shControl + GFP, shEHD1 + GFP-Rab35-WT (shControl + GFP = 100%, shEHD1 + GFP-Rab35-WT = 83.3 ± 12.4%). All graphs from three independent experiments and show mean ± SEM. (ns, not significant; *P < 0.05, **P < 0.01, ***P < 0.001; by unpaired Student’s t-test). All bar graphs were analysed and represented using Graph Pad Prism version 7. Scale bars 10 µm.

The data presented above suggest that Rab35, ACAP2, ARF6-GDP, and EHD1 act along the same pathway in the formation of TNTs. To check whether this was indeed the case, we started by overexpressing GFP-ARF6-T27N in cells in which endogenous EHD1 was depleted by shRNA and measured both the number of TNT-connected cells and the vesicle transfer by flow cytometry. We found no change in the percentage of TNT-connected cells compared to the control (Fig. 5a,b), as well as in vesicle transfer between the cells (Fig. 5c, Fig. S3f). Thus, the increase of TNTs observed upon GFP-ARF6-T27N overexpression requires the presence of EHD1, indicating that EHD1 acts downstream of ARF6-T27N. Next, we performed a similar experiment by overexpressing GFP-ACAP2-WT in EHD1 depleted cells. Like for ARF6-T27N, the overexpression of ACAP2 showed no change in the percentage of TNT-connected cells (Fig. 5d,e), nor in vesicle transfer (Fig. 5f, Fig. S3g) when EHD1 was depleted. This is consistent with the hypothesis that EHD1 acts downstream of ACAP2 and ARF6. Finally, to test the role of Rab35 in this activation cascade we overexpressed its wild type form in EHD1 depleted cells. Again, we observed no significant change in the percentage of TNT-connected cells (Fig. 5g,h) and vesicle transfer (Fig. 5, Fig. S3h). This corroborates our hypothesis that Rab35 is upstream of EHD1 in the pathway of TNT regulation.

**Figure 6:**
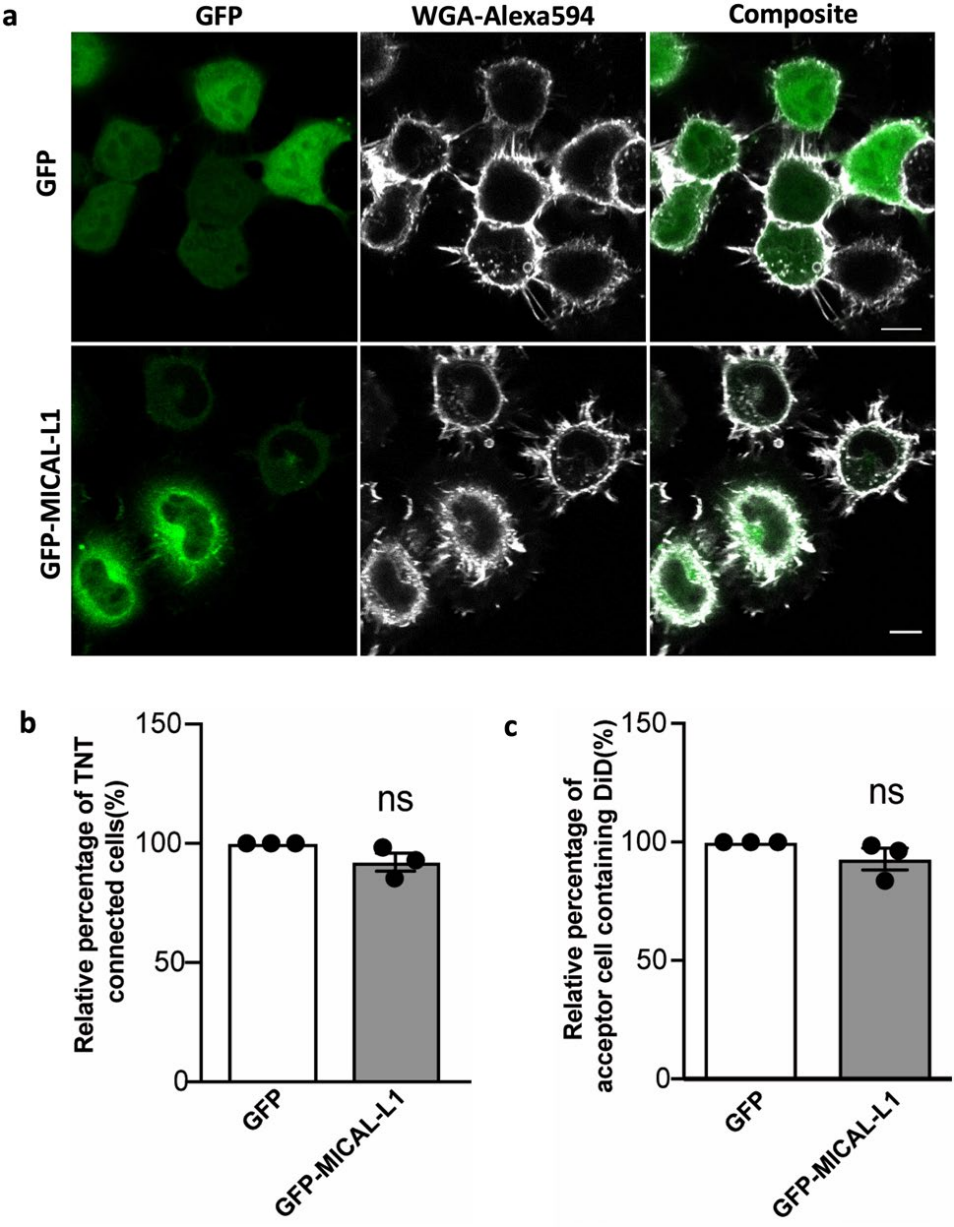
MICAL-L1 has no effect on TNT formation: (**a**) Confocal images of cells transfected with GFP, GFP-MICAL-L1. Arrows indicate TNTs. (**b**) Bar graphs representing the relative percentage of TNT-connected cells described in (a) (GFP = 100%, GFP-MICAL-L1 = 94.5 ± 1.2%). (**c**) Bar graph showing the relative percentage of acceptor cells containing DiD-labelled vesicles from the co-cultures, where donor cells were transfected with GFP, GFP-MICAL-L1 (GFP = 100%, GFP-MICAL-L1 = 95.5 ± 5.5%). All graphs from three independent experiments and show mean ± SEM. (ns, not significant; *P < 0.05, **P < 0.01, ***P < 0.001; by unpaired Student's t-test). All bar graphs were analysed and represented using Graph Pad Prism version 7. Scale bar 10 µm.

This pathway is similar to what was previously shown in neurite outgrowth, where Rab35 recruits both ACAP2 and MICAL-L1 on endosomes, and where MICAL-L1 works along with ACAP2 to increase the formation of neurite protrusions by contributing to directly recruit EHD1^33^. We thus tested whether the overexpression of GFP-MICAL-L1-WT would increase the number of TNTs and transfer of vesicles by using the same assays as described above. Interestingly, no significant effect either on the number of TNT-connected cells (Fig. 6a,b) or vesicle transfer between the cells (Fig. 6c, Fig. S3i) was observed. Thus, this data suggests that Rab35 may regulate TNTs through ACAP2 and ARF6, while it assumes no MICAL-L1 involvement in this pathway. Therefore, our results differentiate the pathway of neurite outgrowth and TNT regulation and strengthen the hypothesis that TNTs are unique protrusions with a distinct formation mechanism.

## Discussion

**Figure 7:**
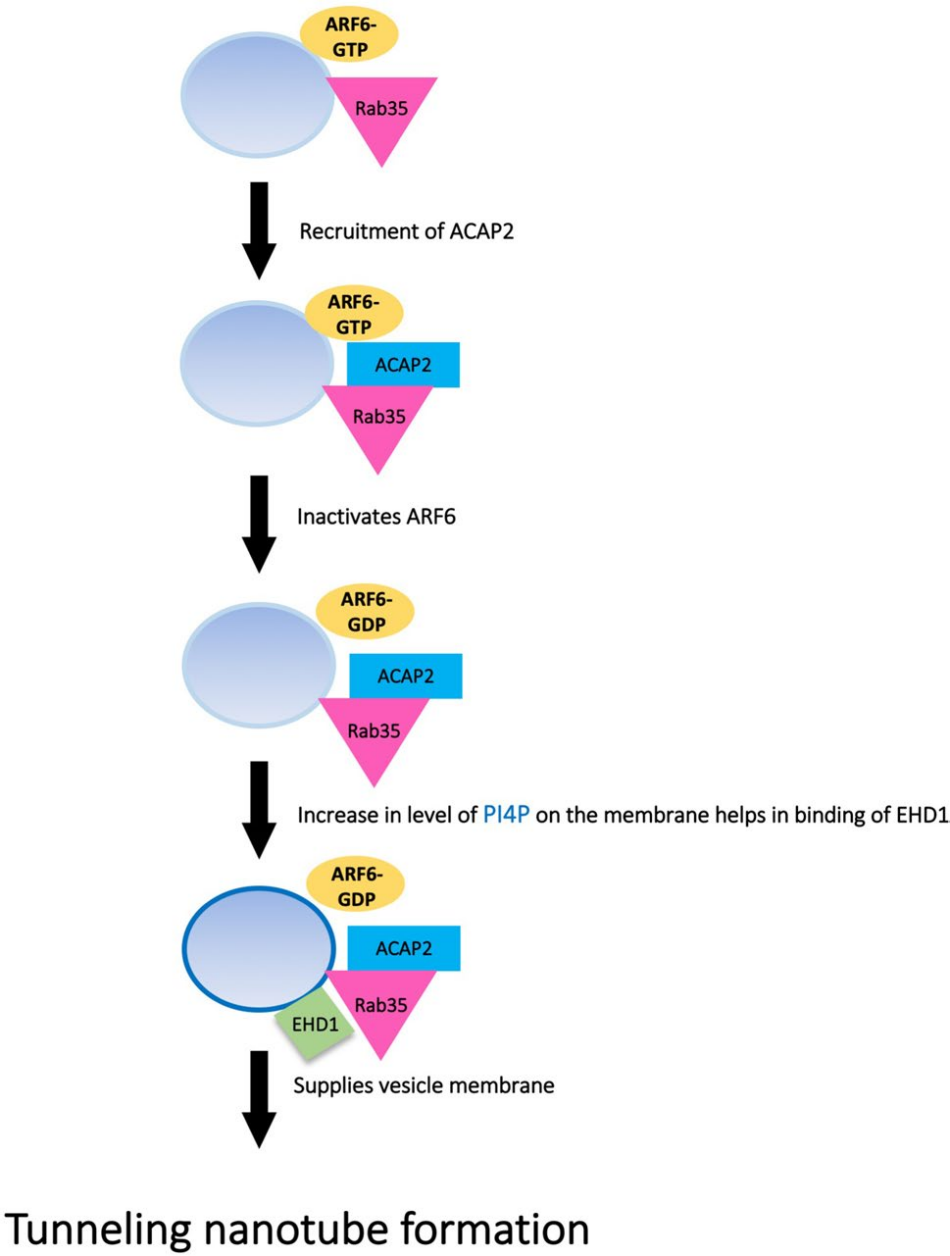
Proposed model of Rab35-dependent TNT formation: ACAP2, a downstream effector of Rab35 and a GAP of ARF6, is recruited by Rab35 to the ARF6- GTP positive vesicular membrane compartments, which inactivates ARF6 thus enabling enrichment of the membrane with PI4P. EHD1 is subsequently recruited to the same compartment due to its affinity for PI4P. This pathway promotes TNT formation, highly likely by supplying the growing TNT with vesicular membrane.

In this study we have established that Rab35-GTP, ACAP2, ARF6-GDP and EHD1 positively regulate TNT formation in neuronal CAD cells. We have also shown that EHD1 is required for TNT formation and acts downstream of Rab35, ACAP2 and ARF6-GDP. We propose that Rab35 and its effector ACAP2 promote TNT formation by inactivating ARF6. The consecutive EHD1 recruitment (see below) then favours the formation of TNTs (Fig. 7). Additionally, we have shown that MICAL-L1 does not participate in the formation of TNTs thus differentiating the mechanism of TNT formation from the one for neurite outgrowth where MICAL-L1, following activation of Rab35, plays an important role. These data uncover new players in TNTs and broaden the currently insufficient knowledge of the role of vesicle trafficking in the field of TNT formation. Furthermore, this study demonstrates that TNTs use a distinct mechanism of formation, reinforcing the hypothesis that they are indeed specific structures different from other cellular protrusions.

Vesicle trafficking was reported to be essential in the process of protrusion formation^32,33^. Though it is intuitive that the cytoskeleton may be one of the major influencers in the formation of neurites, it was shown that the formation of these protrusions is also controlled by vesicle trafficking^62^ and by Rab GTPases which regulate proteins that are directed to the neurite outgrowth^63^. Similar to neurites, in addition to being composed of actin cytoskeleton, TNTs are membranous structures, which raises the question of how membrane is supplied for their growth and what are the key molecular players involved in this process. Previous studies indicated that in addition to actin remodelling, membrane recycling pathways play a role in TNT formation^59^. In support of this hypothesis, we recently reported that inhibition of membrane recycling from the endosomes to the plasma membrane has a negative impact on TNTs, thus implicating the role of vesicle recycling in the formation of TNTs^32^.

Furthermore, a screen of 41 Rab proteins previously conducted in our lab led to the discovery that Rab11a and Rab8a act in a cascade mechanism to stimulate TNT formation through the action of VAMP3 and membrane recycling^32^. Among the others, Rab35, was another strong positive candidate for TNT formation in our screen. As Rab35 is an important regulator of membrane recycling^47,48^, and was shown to be a master regulator of neurite outgrowth^33^, we hypothesize that neurite outgrowth and TNT formation may be related or they might form by employing a similar mechanism.

During neurite outgrowth, Rab35 acts along with ACAP2 to inactivate ARF6 and facilitate the recruitment of EHD1^33^. Since in our study we have found Rab35 to be a positive regulator of TNT formation, and knowing the Rab35 involvement in protrusion formation through vesicle trafficking, we propose that when Rab35 is overexpressed as a wild type or active form in our cells, it operates along the same vesicle recycling pathway to positively regulate TNTs.

Remarkably, Rab35 effectors ACAP2, GDP-bound (inactive) ARF6 and EHD1 were individually shown to induce functional TNTs. In addition, we found that their effect was dependent on the presence of EHD1 which acts as a downstream effector molecule in the pathway (Fig. 7). ACAP2, being a GAP of ARF6, inactivates ARF6, which has been shown to regulate the level of the lipids in the membrane. Specifically, the active form of ARF6 promotes the conversion of PI4P to PI(4,5)P2^61^. Therefore, when ACAP2 inactivates ARF6 we speculate that there should be more PI4P in the membrane of recycling endosomes. EHD1 has an affinity for PI4P over PI(4,5)P2^64^, which might consequently lead to EHD1 recruitment to the membrane compartments rich in PI4P. In our neuronal cells inactive GDP-ARF6 positively influenced the formation of TNTs. Furthermore, activated ARF6 was previously found to indirectly impact actin cytoskeleton by recycling the proteins such as CDC42 and Rac to the cell leading edge to promote cell migration^65^. Considering CDC42 negatively regulates TNTs in CAD neuronal cells^30^, we postulate that in our case ARF6-GDP positively regulated TNTs by recruiting EHD1, but we do not exclude an additional role of ARF6 on CDC42 and actin cytoskeleton that would also affect formation of TNTs.

Interestingly, during neurite outgrowth, EHD1 was found to be recruited to recycling endosomes by two different pathways, on one side through a direct interaction with MICAL-L1, and on the other indirectly through the action of ACAP2 on AFR6^33^. In our hands, MICAL-L1 overexpression had no effect on TNTs, suggesting that it does not have a role in TNT formation. Therefore, we can hypothesize that Rab35 recruits ACAP2, which in turn inactivates ARF6 leading to an increase in PI4P levels, thus enabling the membrane recruitment of EHD1 which then positively regulates TNT formation (Fig. 7). Overall this data demonstrates that even though they use similar pathways through the activation of Rab35 and the recruitment of EHD1, neurite outgrowth and TNT formation are regulated in a different manner. We also show here that this pathway does not affect the number of attached filopodia, thus highlighting the difference between these protrusions and TNTs. This is consistent with a previous study showing that filopodia are structurally different from TNTs^4^ and are differently regulated^30,32^.

How would EHD1 promote TNT formation? Endocytic recycling has been proposed to be crucial to supply membranes and/or proteins to neurite tips and growing protrusions like cilia, filopodia and also TNTs to enable their extension^33,36,54,66,67^. In a previous study we demonstrated that Rab11a and Rab8a act in a cascade upstream of VAMP3 to induce TNTs^32^. In the same study, we showed that by blocking membrane recycling from the endosomes to the plasma membrane using a drug primaquine, there is a subsequent reduction of TNT formation, reinforcing the hypothesis of the involvement of this process also in TNTs like in other protrusions^32^. The mechanism by which EHD1 works for both neurite outgrowth and TNT formation could be the same, through promoting endocytic recycling, and in particular the trafficking from recycling endosomes to the plasma membrane^33,53^. Mechanistically, being a dynamin-like protein which is found on the endocytic recycling compartment, EHD1 enables the fission of PI4P-rich vesicles^68^ which eventually may provide membrane material for TNTs. In accordance with what has been previously published and with the information we obtained in this study, we propose that the mechanism through which EHD1 may facilitate TNT growth in response to active Rab35 is by supplying membrane material by vesicles targeted to the budding TNT from recycling endosomes. Interestingly, EHD1 was found to localize also at the preciliary membranes where, by its involvement in ciliary vesicle tubulation, was shown to initiate a cilium^36^. Albeit our work clearly emphasizes that Rab GTPases and their respective interacting partners, i.e. EHD1, have a crucial role in the process of TNT formation, it would be interesting to elucidate how the vesicles positive for the aforementioned proteins favour the formation of TNTs and whether these proteins are found in its base and/or along its length.

This study provides novel insights into the role of Rab GTPases and recycling endosomes and thus deepens the knowledge of TNT formation but also assigns a new and appealing role to Rab proteins in their already broad pool of functions. This will provide us with a novel direction for the study of molecular effectors involved in this currently poorly understood but highly important process of TNT formation.

## Materials and methods

### Cell lines, plasmids and transfection procedures

The mouse catecholaminergic neuronal CAD cell line was grown in Gibco’s Opti-MEM supplemented with 10% fetal bovine serum (FBS) and 1% Penicillin–Streptomycin, as described elsewhere^26,30,32^. pEGFP-C1-Rab35 (WT), pEGFP-C1-Rab35 (Q67L)—active form, pEGFP-C1-Rab35 (S22N)—dominant negative form, pEGFP-C1-ACAP2 (WT), shRNA non-target control and shRNA against EHD1 were prepared as described previously^33,47^. GFP-MICAL-L1 (WT), pEGFP-ARF6 (WT), pEGFP-ARF6 (Q67L)—active form and pEGFP-ARF6 (T27N)—inactive form were a gift from Arnaud Echard. GFP-EHD1 (WT) was a gift from Christopher Westlake (National Institutes of Health, USA). GFP-vector, mCherry-H2B and EBFP-H2B were purchased from Addgene. CAD cells were transiently transfected with Lipofectamine 2000 (Invitrogen, Thermo Fisher Scientific) according to the manufacturer’s instructions and as previously reported^26^.

### Labelling and quantification of TNT-connected cells

Confluent CAD cells were mechanically detached and counted, and 300,000 cells were plated for 6 h per well in a 6 well plate. Cells were transfected as described above, using the abovementioned plasmids. At 24 h post-transfection, cells were detached and counted, and 200,000 cells were plated for 16 h on ibidi μ-dishes (ibidi GmbH). After 16 h post-seeding, cells were fixed with fixative solution 1 (2% PFA, 0.05% glutaraldehyde and 0.2 M HEPES in PBS) for 20 min at 37 °C followed by a second fixation for 20 min with fixative solution 2 (4% PFA and 0.2 M HEPES in PBS) at 37 °C. The cells were gently washed with PBS and labelled with WGA-Alexa 594 (Invitrogen, Thermo Fisher Scientific) (1:300 in PBS) for 20 min at room temperature, and/or labeled with Rhodamine Phalloidin (Invitrogen, Thermo Fisher Scientific) (1:300 in PBS) for 30 min, washed with PBS three times before staining with DAPI (1:1000 in PBS) for 2 min and subsequently washed with PBS prior to being sealed with Aqua Poly/Mount (Polysciences, Inc.), as previously described^30,32,60^.

Image stacks (of 0.4 µm slice thickness) covering the whole cellular volume were acquired using a confocal microscope (Zeiss LSM 700) controlled by ZEN software. To evaluate the number of TNT-connected cells, manual analysis was performed in ICY software (https://icy.bioimageanalysis.org/) using the ‘Manual TNT annotation’ plugin by counting the transfected cells containing a TNT, as described elsewhere^26,32,60^.

The two cells which were connected with at least one continuous connection were marked as TNT-connected cells. TNT-connected cells were assessed and quantified only in the middle and upper stack; first 5 slices were excluded from the analysis to avoid counting substrate-attached protrusions (Movie S1). At least 50 transfected green cells were counted for each sample per each individual experiment, having in total at least 150 cells analysed. To quantify TNTs following transfection, only TNTs found between two transfected cells, and between one transfected and one non-transfected were counted; TNTs formed between two non-transfected cells were not taken into consideration. Image analyses and displays of raw data, such as Z-projections, were obtained using ICY software and ImageJ^60^. Z-stack animation (Movie S1) was processed in ImageJ and Adobe Premiere Pro.

### Attached filopodia detection and quantification

As described before^20^, confluent CAD cells were mechanically detached and counted, and 300,000 cells were plated for 6 h per well in a 6 well plate. Cells were transfected as described above, using the abovementioned plasmids. At 24 h post-transfection, cells were detached and counted, and 140,000 cells were plated for 16 h on ibidi μ-dishes (ibidi GmbH). Cells were then fixed with 4% PFA for 20 min at RT and washed three times with PBS. Cells were then incubated for permeabilization and blocking with 2% BSA including 0.0075% saponin at RT for 1 h. Primary monoclonal antibody of vinculin (Sigma, 1:500) was prepared in PBS having 2% BSA and 0.01% saponin and incubated at RT for 1 h. After several washes with PBS cells were incubated with secondary antibody goat anti-mouse Alexa Fluor 546 (Invitrogen, Thermo Fisher Scientific) in the same solution at RT for 1 h. Cells were then stained with HCS Cell Mask Blue Stain (Invitrogen, Thermo Fisher Scientific, 1:5000) in PBS for 20 min, washed several times and mounted. Quantification was performed as described before^20,30,32^ by (1) creating the ROI restricted to the outer region of the cells that covers only attached filopodia; (2) automatized counting of the vinculin positive filopodia using spot detector tool (ICY software).

### Fluorescence microscopy to image the transfer of DiD-labelled vesicles

As described elsewhere^32^, confluent CAD cells were mechanically detached and counted and 800,000 cells were plated for 6 h in T25 flasks. The cells were transfected with the appropriate GFP tagged constructs for donor cells and EBFP-H2B (a histone protein fused with a fluorescence tag used to stain nuclei in live cells) for acceptor cells for 24 h in complete medium. The donor cells were labelled with a 333 nM solution of the lipophilic tracer Vybrant DiD (Thermo Fisher Scientific) in complete medium for 30 minn at 37 °C. Cells were then washed with PBS and 0.01% trypsin, subsequently resuspended in complete medium and counted. The labelled donor cells were mixed in a 1:1 ratio with H2B-transfected acceptor cells and plated at subconfluence (220,000 cells) on ibidi μ-dishes (ibidi GmbH) for 16 h at 37 °C. Cells were fixed with fixative solution 1 (2% PFA, 0.05% glutaraldehyde and 0.2 M HEPES in PBS) for 20 min at 37 °C followed by a second 20 min fixation with fixative solution 2 (4% PFA and 0.2 M HEPES in PBS) at 37 °C. Co-culture cells were labeled with Rhodamine Phalloidin (Invitrogen, Thermo Fisher Scientific) (1:300 in PBS) for 30 min to visualize actin and TNTs in the co-culture. Samples were washed and sealed with Aqua Poly/Mount (Polysciences, Inc.). The cells were then imaged with an inverted confocal microscope (Zeiss LSM 700) controlled by ZEN software and images were processed in ImageJ.

### Flow cytometry to analyse the transfer of DiD-labelled vesicles

Confluent CAD cells were mechanically detached and counted and 800,000 cells were plated for 6 h in T25 flasks. Cells were transfected with the appropriate GFP tagged constructs for donor cells and with H2B-mCherry for acceptor cells, for 24 h in complete medium. For the knockdown experiments, cells were plated at 400,000 cells in 60 mm dishes for 6 h and then transfected with shRNA. After 16 h they were transfected with shRNA together with GFP-tagged constructs for 24 h in complete medium. Donor cells were detached, counted and labelled with a 333 nM solution of the lipophilic tracer Vybrant DiD (Thermo Fisher Scientific) in complete medium for 30 min at 37 °C. Cells were then washed with PBS and 0.01% trypsin, subsequently resuspended in complete medium and counted, as stated elsewhere^30,32^.

The labelled donor cells were mixed in a 1:1 ratio with H2B-mCherry transfected acceptor cells and plated at subconfluence (200,000 cells per well) on 24-well plates for 16 h at 37 °C. Cells were then washed with PBS, mechanically detached from the dish by pipetting up and down with 500 μl PBS and passed through sterile 40 μm nylon cell strainers (BD Falcon) in order to obtain single-cell suspensions. Cell suspensions were fixed with 500 μl of 4% PFA (2% final solution), as previously described^30,32^. A ‘supernatant’ control was performed to verify that the transfer of vesicles between cells is mainly cell–cell dependent and not secretion-based. After overnight culturing, conditioned medium from donor cells was centrifuged at 1000 rpm for 5 min to remove cell debris and preserve the vesicles, and was then transferred to the acceptor cells for an additional 16 h of culture (Fig. S2). An additional ‘mixture’ control was added by mixing samples of separate donor and acceptor cells after fixation to remove any possible false positive results due to sample preservation in the fixative. Both of these controls were subtracted from the total transfer in coculture to obtain cell–cell dependent transfer represented in the final figures. Flow cytometry data were acquired using an LSR Fortessa flow cytometer (BD Biosciences). GFP fluorescence was analysed at 488 nm excitation wavelength, mCherry fluorescence was analysed at a 561 nm excitation wavelength and DiD fluorescence was analysed at a 640 nm excitation wavelength. Samples were analysed at a high flow rate, corresponding to 200–400 events per second, and at least 10,000 events were acquired for each condition, as previously stated^30,32^. The data was analysed using FlowJo analysis software.

### Western blot

Cells transfected with shRNA were lysed in NP-40 lysis buffer (25 mM Tris, pH 7–8, 150 mM NaCl, 0.1% SDS, 0.5% sodium deoxycholate, 1% Triton X-100), and protein concentration in the cell lysate was quantified using a Bradford protein assay (Bio- Rad), as previously described^30^. Protein samples were incubated at 100 °C for 5 min and electrophoresed on 10% SDS–polyacrylamide gels. Proteins were transferred to PVDF membranes (GE Healthcare Life Sciences). Membranes were blocked in 5% non-fat milk in Tris- buffered saline with 0.1% Tween 20 (TBS-T) for 1 h. Membranes were then incubated at 4 °C with following primary antibody rabbit anti-EHD1 (24657-1-AP, Proteintech), mouse anti-α-tubulin (T5168, Sigma) diluted in 5% non-fat milk overnight (1:500 and 1:10,000, respectively) then washed several times with TBS-T. After 1-h incubation with horseradish peroxidase-conjugated with respective IgG secondary antibody (1:10,000) (GE Healthcare Life Sciences), membranes were washed with TBS-T and protein bands on the membrane were detected using an ECL-Plus immunoblotting chemiluminescence system (GE Healthcare Life Sciences). Membranes were imaged using ImageQuant LAS 500 camera (GE Healthcare Life Sciences), as previously described^30^.

### Statistical analysis

All column graphs and statistical analysis were performed using GraphPad Prism version 7 software. Specific statistical tests used are indicated in each figure legend.

## Supplementary information

Supplementary video.

Supplementary video legend.

Supplementary figures.
