# Supplementary material for: Rab35 and its effectors promote formation of tunneling nanotubes in neuronal cells
Source: Sci Rep. 2020 Oct 8;10:16803. doi: 10.1038/s41598-020-74013-z (PMC7544914; doi:10.1038/s41598-020-74013-z)
Supplement: Supplementary file 2 — Supplementary video legend. [file 41598_2020_74013_MOESM2_ESM.docx]

**Movie S1.** TNT quantification approach. Z-stack (21 slices) movie demonstrating TNT quantification approach, where TNTs are counted in the middle and upper z-stack; first 5 slices are omitted from the quantification to avoid counting protrusions adherent to the substrate. Cells connected via TNTs are labeled with a green circle and connected with a green line to indicate TNT presence, while cells containing no TNTs are labeled with an orange circle. The percent of TNT-connected cells is calculated by dividing the amount of TNT-connected cells with the total amount of cells per image. Cells were labeled with rhodamine-phalloidin to stain for actin and enable TNT-recognition and quantification. Scale bar: 20 μm.
