# Supplementary material for: Rab35 and its effectors promote formation of tunneling nanotubes in neuronal cells
Source: Sci Rep. 2020 Oct 8;10:16803. doi: 10.1038/s41598-020-74013-z (PMC7544914; doi:10.1038/s41598-020-74013-z)
Supplement: Supplementary file 3 — Supplementary figures. [file 41598_2020_74013_MOESM3_ESM.docx]

## Title: Rab35 and its effectors promote formation of tunneling nanotubes in neuronal cells

Shaarvari Bhat^1,2,#^, Nina Ljubojevic^1,3,#^, Seng Zhu^1^, Mitsunori Fukuda^4^, Arnaud Echard^5^ and Chiara Zurzolo^1,*^

1 Unit of Membrane Traffic and Pathogenesis, UMR3691 CNRS, F-75015, Institut Pasteur, 28 rue du Dr Roux, 75015 Paris, France.

2 Université Paris-Sud, Université Paris-Saclay, 91405 Orsay, France.

3 Sorbonne Université, ED394 - Physiologie, physiopathologie et thérapeutique, 75005 Paris, France.

4 Department of Integrative Life Sciences, Graduate School of Life Sciences, Tohoku University, Aobayama, Aoba-ku, Sendai, Miyagi 980-8578, Japan.

5 Membrane Traffic and Cell Division Lab, UMR3691 CNRS, F-75015, Institut Pasteur, Paris, France.

# These authors contributed equally

* Correspondence should be addressed to Chiara Zurzolo

(email: chiara.zurzolo@pasteur.fr)

**Figure S1.** TNTs contain actin and DiD-stained vesicles. Confocal images showing TNTs containing actin and DiD-stained vesicles in GFP, GFP-Rab35-WT, GFP-ACAP2, GFP-ARF6-T27N, GFP-EHD1-transfected cells. Upper slices of acquired z-stack were shown in each condition. Arrows indicating TNTs and, where present, DiD vesicles within TNTs. Scale bars: 10 µm.

**Figure S2.** Schematics representing the co-culture experiment. a) Total transfer is analyzed in a co-culture system where donors express GFP-tagged protein of interest and contain DiD-stained vesicles, and where acceptor cells are transfected with mCherry-H2B nuclei tag to differentiate them from the donors. Donors containing DiD and acceptors are mixed in 1:1 ratio and co-cultured for 16 hours, subsequently fixed and analysed by flow cytometry. b) Supernatant control that corresponds to secretion-based transfer was performed by culturing donors and acceptors separately and then adding the conditioned media from the donors to the acceptors that are further cultured for 16 hours, fixed and analyzed by flow cytometry. c) Post-fixation mixture control is undertaken to eliminate potential post-fixation transfer. Donors and acceptors are mixed after fixation in the same amount as for co-culture, and the mixture is then analyzed by flow cytometry.

 **Figure S3.** DiD-stained vesicles transfer from donors to acceptors mainly by cell-cell contact. **Left panel:** a) Dot plots from a representative co-culture experiment showing the transfer of DiD-stained vesicles from GFP-Rab35 and its mutants donors to the mCherry-H2B acceptors. b) Dot plots from a representative co-culture experiment showing the transfer of DiD-stained vesicles from GFP-ACAP2 donors to the mCherry-H2B acceptors. c) Dot plots from a representative co-culture experiment showing the transfer of DiD-stained vesicles from GFP-ARF6 and its mutants donors to mCherry-H2B acceptors. d) Dot plots from a representative co-culture experiment showing the transfer of DiD-stained vesicles from GFP-EHD1 donors to mCherry-H2B acceptors. e) Dot plots from a representative co-culture experiment showing the transfer of DiD-stained vesicles from shEHD1 + GFP/GFP-EHD1 donors to mCherry-H2B acceptors. f) Dot plots from a representative co-culture experiment showing the transfer of DiD-stained vesicles from shEHD1 + GFP-ARF6-T27N/GFP-ACAP2/GFP-Rab35-WT donors to mCherry-H2B acceptors. g) Dot plots from a representative co-culture experiment showing the transfer of DiD-stained vesicles from GFP-MICAL-L1 donors to mCherry-H2B acceptors. All dot plots were analyzed and represented using FlowJo software.

**Right panel:** corresponding graph bars for supernatant and mixture controls that were normalized to the total control transfer. a) For GFP-Rab35 and its mutants (GFP(supernatant) = 7.2 ± 1.1%, GFP(mixture) = 6.6 ± 1.3%, GFP-Rab35-WT(supernatant) = 7.3 ± 1.7%, GFP-Rab35-WT(mixture) = 5.3 ± 1.2%, GFP-Rab35-Q67L(supernatant) = 8.7 ± 2.8%, GFP-Rab35-Q67L(mixture) = 7.6 ± 1.5%, GFP-Rab35-S22N(supernatant) = 5.5 ± 1.3%, GFP-Rab35-S22N(mixture) = 6.4 ± 2.1%). b) For GFP-ACAP2 (GFP(supernatant) = 6.5 ± 1.7%, GFP(mixture) = 7.7 ± 1.2%, GFP-ACAP2(supernatant) = 6.5 ± 0.8%, GFP-ACAP2(mixture) = 7.0 ± 2.0%). c) For GFP-ARF6 and its mutants (GFP(supernatant) = 7.4 ± 0.7%, GFP(mixture) = 7.0 ± 1.4%, GFP-ARF6-WT(supernatant) = 9.1 ± 1.2%, GFP-ARF6-WT(mixture) = 7.0 ± 0.6%, GFP-ARF6-Q67L(supernatant) = 10.5 ± 4.0%, GFP-ARF6-Q67L(mixture) = 5.9 ± 0.3%, GFP-ARF6-T27N(supernatant) = 9.0 ± 1.2%, GFP-ARF6-T27N(mixture) = 6.6 ± 1.0%). d) For GFP-EHD1 (GFP(supernatant) = 3.8 ± 0.5%, GFP(mixture) = 4.5 ± 0.3%, GFP-EHD1(supernatant) = 4.4 ± 0.9%, GFP-EHD1(mixture) = 7.2 ± 1.9%). e) For shEHD1 (shControl+GFP(supernatant) = 8.9 ± 3.6%, shControl+GFP(mixture) = 2.6 ± 0.6%, shEHD1+GFP(supernatant) = 5.5 ± 2.4%, shEHD1+GFP(mixture) = 2.1 ± 1.3%, shEHD1+GFP-EHD1(supernatant) = 5.8 ± 2.4%, shEHD1+GFP-EHD1(mixture) = 4.2 ± 1.3%). f) For shEHD1+GFP-ARF6-T27N (shControl+GFP(supernatant) = 5.1 ± 1.3%, shControl+GFP(mixture) = 4.9 ± 1.3%, shEHD1+GFP-ARF6-T27N(supernatant) = 5.1 ± 1.8%, shEHD1+GFP-ARF6-T27N(mixture) = 3.8 ± 0.4%). g) For shEHD1+GFP-ACAP2 (shControl+GFP(supernatant) = 4.9 ± 1.0%, shContro|+GFP(mixture) = 4.4 ± 1.6%, shEHD1+GFP-ACAP2(supernatant) = 4.8 ± 1.5%, shEHD1+GFP-ACAP2(mixture) = 5.9 ± 1.1%). h) For shEHD1+GFP-Rab35-WT (shControl+GFP(supernatant) = 9.4 ± 2.8%, shContro|+GFP(mixture) = 4.1 ± 1.3%, shEHD1+GFP-Rab35-WT(supernatant) = 8.5 ± 1.8%, shEHD1+GFP-Rab35-WT(mixture) = 5.5 ± 1.7%). i) For GFP-MICAL-L1 (GFP(supernatant) = 5.0 ± 0.4%, GFP(mixture) = 5.3 ± 0.5%, GFP-MICAL-L1(supernatant) = 6.7 ± 0.4%, GFP-MICAL-L1(mixture) = 5.7 ± 0.6%). All bar graphs were analysed and represented using Graph Pad Prism version 7.

**Figure S4.** DiD-stained vesicles transfer from donors overexpressing Rab35 and its effectors to EBFP-H2B acceptors. Confocal images representative of co-culture vesicle transfer, where donors are transfected with GFP-tagged protein containing DiD-stained vesicles in co-culture with EBFP-H2B-acceptors. Co-culture stained with rhodamine-phalloidin after fixation to visualize the cells and actin-containing TNTs. Arrows indicating DiD vesicles present in the acceptor cells, and where detected in the upper stack, TNTs connecting donor with acceptor cell. Scale bars: 10 µm.

**Figure S5:** shEHD1 depletes endogenous EHD1 in CAD cells. Full blot of shEHD1 depletion showing effective knock-down of EHD1 after 48 and 72 hours of shEHD1.

**Figure S6:** ARF6-GDP, Rab35, ACAP2 and EHD1 have no impact on filopodia:
a) Confocal images of cells transfected with GFP, GFP-ARF6-T27N, GFP-Rab35-WT, GFP-ACAP2 and GFP-EHD1 and stained for vinculin. b) Bar graphs representing the average number of attached filopodia per cell described in (a) (GFP = 44.4 ± 2.4%, GFP-ARF6-T27N = 41.9 ± 5.2%, GFP-Rab35-WT = 43.9 ± 2.6%, GFP-ACAP2 = 45.0 ± 6.7%, GFP-EHD1 = 48.6 ± 6.9%). All graphs from three independent experiments and show mean ± SEM. (ns, not significant; *P<0.05, **P<0.01, ***P<0.001; by unpaired Student's t-test). All bar graphs were analysed and represented using Graph Pad Prism version 7. Scale bars: 10 µm.
